# Carbon aerogels with improved flexibility by sphere templating†

**DOI:** 10.1039/c8ra04848g

**Published:** 2018-07-31

**Authors:** Miralem Salihovic, Nicola Hüsing, Johannes Bernardi, Volker Presser, Michael S. Elsaesser

**Affiliations:** Chemistry and Physics of Materials, University of Salzburg 5020 Salzburg Austria michael.elsaesser@sbg.ac.at +43-662-8044-6262; USTEM, Vienna University of Technology 1040 Vienna Austria; INM – Leibniz Institute for New Materials 66123 Saarbrücken Germany; Saarland University 66123 Saarbrücken Germany

## Abstract

Mechanically reversible compressible resorcinol–formaldehyde (RF) aerogels can be converted into mechanically reversible compressible carbon aerogels (CA) by carbonization in an inert atmosphere. By incorporation of polystyrene spheres into the RF gels as a sacrificial template, it is possible to create macropores with controlled size within the carbon framework during carbonization. The resulting templated carbon aerogel shows enhanced mechanical flexibility during compression compared to pristine samples. In addition, the presence of hierarchical porosity provides a porous architecture attractive for energy storage applications, such as supercapacitors.

## Abstract 2

Using soft templating, mechanically reversible compressible resorcinol–formaldehyde aerogels can be converted into mechanically reversible compressible carbon aerogels with high surface area by carbonization in an inert atmosphere.

## Graphical abstract

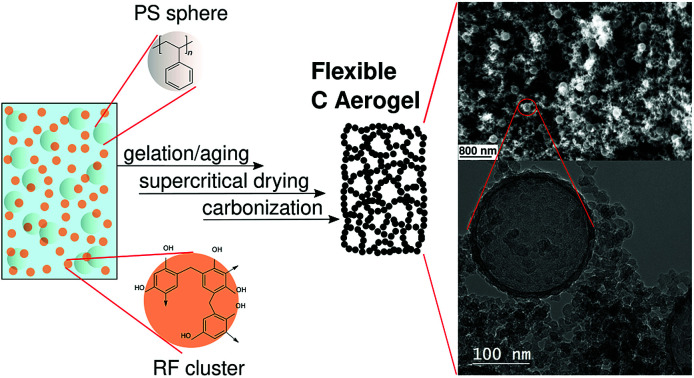

## Introduction

Carbon aerogels (CAs) are fascinating solids with a unique combination of material properties, such as electrical and thermal conductivity, low density, and high surface area.^1,2^ Accordingly, CAs are attractive for many applications, including supercapacitors,^3^ thermal insulation,^4^ and filtration/separation.^5^ Typical CAs are fragile, and this severely limits the current range of applications.^6,7^ To overcome this issue, mechanically reversible compressible carbon aerogels can be used and have been demonstrated for recyclable oil absorption^8^ and flexible electrode materials.^9^ A reversibly compressible CA tolerates volume expansion phenomena like pore swelling or ion intercalation without destruction to a much higher extent than a stiff material.^10^ To the best of our knowledge, only a few studies have reported reversibly compressible carbon aerogels so far; for example, CA-based on graphene,^9,11,12^ carbon nanotubes (CNTs),^4^ bacterial cellulose,^6^ biomass,^13^ nanofibers,^14^ or resorcinol–formaldehyde (RF).^7^ For RF-based CAs, a low resorcinol concentration in water combined with precise control of the pH value enables the synthesis of mechanically reversibly compressible RF gels^15^ which can be converted into the respective porous carbon analogues.^16^ The mechanical or electrochemical properties of CAs can further be modified, for example, by incorporation of CNTs,^17,18^ silica,^19^ or metal particles.^20^

Recently, Schwan, and Ratke showed by *in situ* compression in a scanning electron microscope that macropores deform elastically and contribute the most to the compressive modulus.^7^ Building on this concept, we introduce an effective approach to control the magnitude of the compressive modulus by controlling the number and the size of templated macropores. We used polystyrene (PS) as a template for macropores in the filigree materials network to obtain mechanically reversible compressible carbon aerogels. The number and size of the pores can easily be tailored by variation of the amount and size of the PS spheres, which are converted to hollow spheres by thermal treatment.^7,21,22^ This templating strategy allows to manipulate the network build-up of the carbon aerogel and with respect to its mechanical properties the compression modulus and the mechanical robustness. Our work also shows that the material can be used for electrochemical energy storage by capitalizing on the electrical conductivity and high surface area.

## Experimental

### Materials and synthesis

We used monodisperse polystyrene (PS) spheres with a diameter of 210 ± 6 nm, as determined by image analysis of scanning electron micrographs (the corresponding average particle diameter was 244 nm determined by dynamic light scattering). The PS spheres were prepared by emulsion polymerization of styrene using potassium persulfate (KPS) as the initiator and polyvinylpyrrolidone (PVP) as the stabilizer, as previously reported.^23^ A mass of 0.1 g of PS spheres was added to a resorcinol formaldehyde (RF) sol which was prepared according to ref. 15. For comparison, pristine RF samples were also prepared without PS addition. The sol–gel reaction of resorcinol with formaldehyde was catalyzed by sodium carbonate (Na_2_CO_3_; R/C = 50), and we adjusted the pH value to 5.45 by adding diluted nitric acid (HNO_3_) for condensation. We obtained cylindrical monolithic RF aerogels samples after gelation and aging at 80 °C for seven days. The RF aerogels were immersed in an acetone bath (50 mL) and the liquid was exchanged and replenished three times in three days. Wet RF aerogels were dried with supercritical CO_2_ in an autoclave by Parr Instruments with a volume of 300 mL at 9 MPa and 55 °C. Subsequently, the RF samples were carbonized in a tube furnace at 800 °C under argon gas.

### Material characterization

The morphologies of RF and carbon aerogels were analyzed with a ZEISS Ultra Plus scanning electron microscope (SEM) using an in-lens detector. The acceleration voltage was adjusted between 2 kV to 5 kV. A thin layer of gold was sputtered onto the RF samples to reduce electrical charging on the sample surface. Transmission electron microscope (TEM) images were recorded with a TECNAI F20 field emission electron microscope using a Gatan Orius SC600 CCD camera. An accelerating voltage of 200 kV was used. TGA measurements were carried out with a NETZSCH STA 449 F3 Jupiter instrument from 20 °C to 1000 °C with a heating rate of 10 °C min^−1^ using an argon or oxygen atmosphere. Nitrogen sorption isotherms were recorded with a Micromeritics ASAP 2420 sorption system at −196 °C. The samples were degassed at 300 °C for 12 h. Specific surface areas (SSA) and pore size distribution were calculated by use of the quenched solid density functional theory (QSDFT). Dynamic light scattering (DLS) and zeta potential measurements were conducted on a Malvern Zetasizer instrument. Data were recorded at a light-backscattering angle of 173°. Each measurement encompassed 30 separate DLS measurements each with 3 sub-runs of 10 s duration. The bulk density of a carbon aerogel sample was defined by the ratio of the mass to the volume. The mass was measured with an ALC microscale by ACCULAB and the monolith's volume was assumed to be perfectly cylindrical.

### Mechanical testing

Compression testing was carried out on a uniaxial universal testing machine (Zwick/Roell Z-250) at the Salzburg University of Applied Sciences' campus in Kuchl. The machine parameters were set at 5 mm min^−1^ compression rate with a 1 kN load cell. A typical compression test consists of a flexibility test (five cycles of compression up to 10% strain) and a stability test (one single compression up to 50% strain) which is depicted in Fig S1.† The universal testing machine recorded force on a sample and its change in length. Force was converted to stress according to eqn (1):1
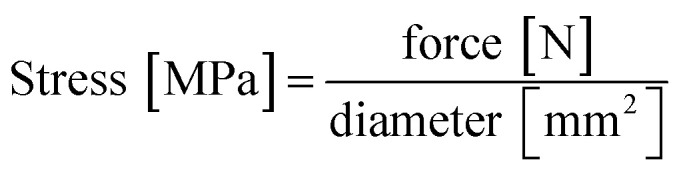


The change in length was converted to strain by dividing the value with the initial length (eqn (2)):2
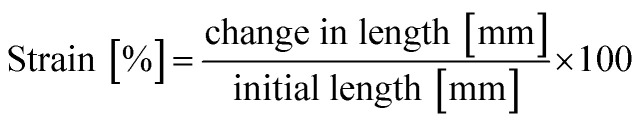


The Young's modulus was calculated by fitting a straight line onto a linear segment in the stress/strain curve approximately between 2% and 8% strain. The slope corresponds to the Young's modulus (eqn (3)):3
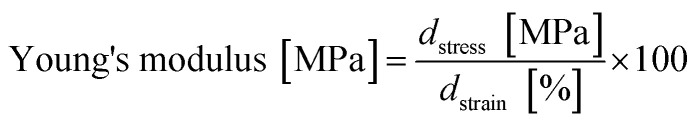


### Electrochemical characterization

Electrochemical characterization was carried out after crushing the material in a mortar and admixing 5 mass% of PTFE to consolidate free-standing electrodes. All measurements were done in half-cell configuration with an oversized activated carbon counter electrode and an Ag/AgCl reference electrode. The cell design can be found in ref. 24 and we used an aqueous 1 M NaCl electrolyte.

## Results and discussion

**Fig. 1:**
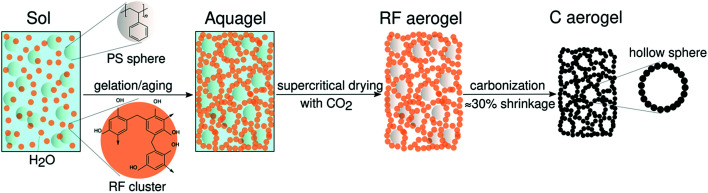
Synthesis scheme for reversibly compressible carbon aerogels with improved mechanical stability.

The synthesis of reversibly compressible carbon aerogels with enhanced mechanical stability was achieved by carbonization of monolithic RF/PS spheres composite gels as outlined in Fig. 1. The diameter of the PS spheres was 210 ± 6 nm according to SEM evaluation (see ESI, Fig. S2†). The sphere diameter determined by DLS was 244 nm (ESI, Fig. S3†). The sol–gel processing of the liquid RF precursor solution mixed with PS spheres leads to a homogeneous dispersion of the latter within the RF polymer network. We used supercritical carbon dioxide drying of these organogels to minimize shrinkage of the network. By this way, we obtained an aerogel instead of a xerogel. Subsequent carbonization of the RF/PS composite aerogels caused the thermal decomposition of the PS spheres, resulting in the formation of hollow spheres throughout the final monolithic carbon aerogel network.

**Fig. 2:**
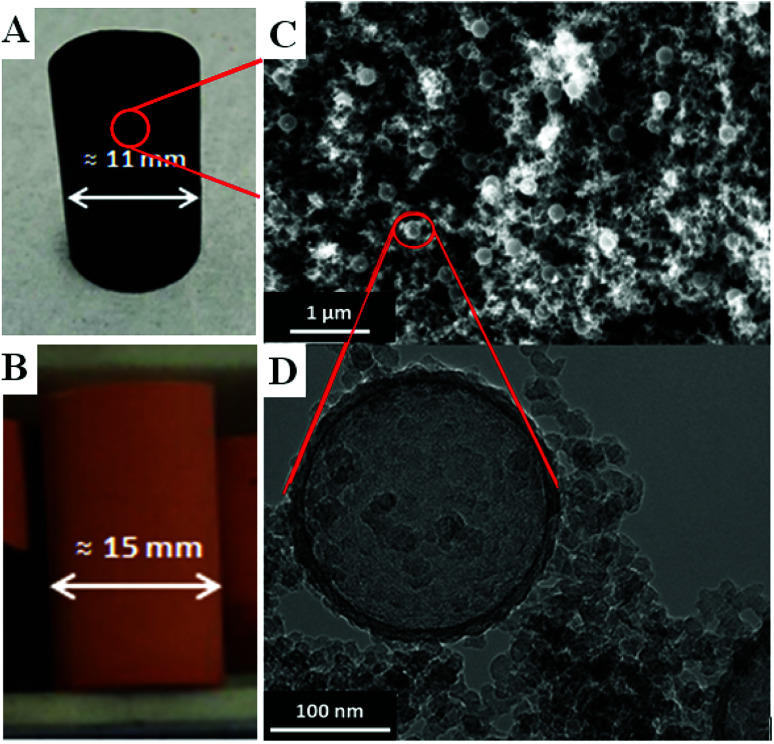
Photographs of (A) flexible carbon aerogel with incorporated hollow spheres and (B) the corresponding reversibly compressible RF gel (precursor with embedded PS spheres) prior to pyrolysis. (C) Scanning electron micrograph and (D) transmission electron micrograph of the modified carbon aerogel.

Scanning and transmission electron micrographs of the skeletal carbon aerogel structure are shown in Fig. 2. As can be seen, the carbon aerogel network consists of interconnected globular RF particles with a diameter of about 10 nm. The homogeneously dispersed PS spheres seem to serve as nucleation sites for the formation of the RF gel network structure. Therefore, we propose a formation mechanism in two stages: First, the attachment of RF clusters/particles onto the negatively charged PS surface takes place (see also the Zeta potential data in the ESI, Fig. S4† ). Second, there occurs covalent bonding of the PS spheres coated with RF clusters into a three-dimensional network.

In this study, we prepared cylindrical monolithic samples with a diameter of 1 cm and 2–3 cm in height (Fig. 2). Pristine flexible RF gels were prepared from a sol with a molar ratio of resorcinol to water (R/W) of 0.008, a molar ratio of resorcinol to formaldehyde (R/F) of 0.5, a molar ratio of resorcinol to sodium carbonate of 50, and a pH value of 5.45 at the beginning of condensation. The sol was stirred for 1 h before pouring it into glass vessels for gelation and subsequent aging at 80 °C for seven days. After a solvent exchange with acetone, the RF gels were dried supercritically with CO_2_ (*P*_c_ = 7.4 MPa and *T*_c_ = 31.1 °C), and subsequent carbonization was performed at 800 °C in an argon atmosphere. In the case of PS modified samples, we added a suspension of monodisperse PS spheres (PdI = 0.020) in deionized water (ESI, Fig. S2 and S3†) to the RF sol after 1 h.

The supercritically dried RF/PS composite gel appears brighter orange colored in comparison to the pristine RF gel (ESI, Fig. S5†). As PS decomposes during carbonization, the PS spheres become hollow with a wall thickness of approximately 10 nm, they retain 95% of the initial diameter, and are embedded in the globular carbon network (ESI, Fig. S6†). We confirmed the hollow character by using scanning transmission electron microscopy (STEM; ESI, Fig. S7†). While the sphere diameter is largely maintained, we observed a macroscopic shrinkage of the monolith from RF to C of about 30% after carbonization. However, we observe a higher decrease in bulk density for samples containing PS regarding the transition from RF to carbon (Table 1). While the density increases slightly after carbonization in case of PS-free samples, we obtain a density decrease of 15% due to PS decomposition.

**Table 1:** Mechanical and physical properties of reversibly compressible RF and C aerogel samples with and without PS addition. *ρ*_bulk_: bulk density; *E*_C_: compressive modulus; *ε*_max_: maximum compressive strain; SSA: specific surface area (QSDFT); PV: pore volume at 95% relative pressure

Sample	*ρ*_bulk_ (g cm^−3^)	*E*_C_ (MPa)	*ε*_max_ (%)	SSA (m^2^ g^−1^)	PV (cm^3^ g^−1^)
RF	0.064	0.08	10	<50	<10
RF + PS	0.066	0.03	10	<50	<10
C	0.070	0.31	30	577	0.43
C + PS	0.056	0.16	>50	661	0.44

**Fig. 3:**
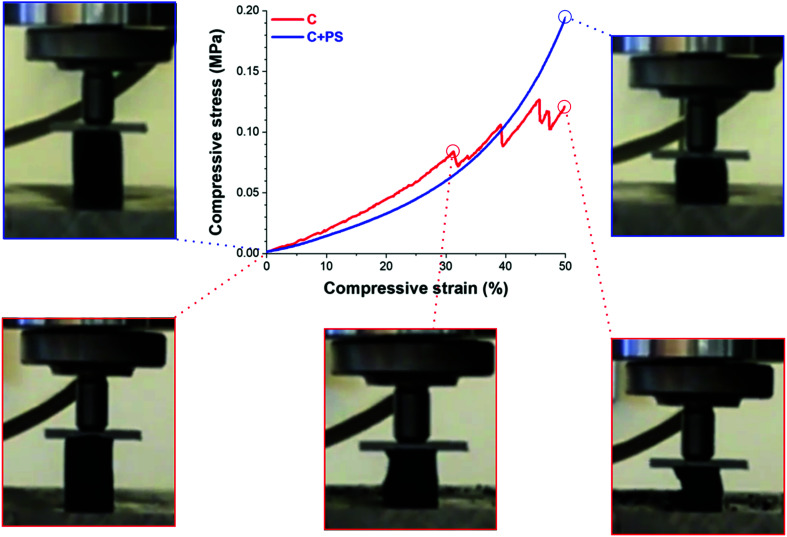
Compression test (stress–strain) of reversibly compressible carbon aerogels with and without PS addition.

The mechanical properties of the aerogel samples were investigated by uniaxial compression tests, and the results are shown in Table 1 and Fig. 3. The RF gels exhibit a low compressive modulus *E*_c_ in the range of 0.03 MPa to 0.08 MPa. The corresponding carbon aerogels show an increase of *E*_c_ with 0.31 MPa for samples without hollow spheres and 0.16 MPa for samples with hollow spheres. The corresponding bulk densities reflect the mechanical behavior: PS addition to the RF gel increases the aerogel density from 0.064 g cm^−3^ to 0.070 g cm^−3^ while the density decreases by 20% for the carbon analogue due to the decomposition of PS from 0.066 g cm^−3^ to 0.056 g cm^−3^. A similar linear density/compressive modulus behavior for highly porous materials is described by Gibson & Ashby^25^ and Pekala *et al.*^26^ Specific conditions of supercritical drying may affect the density of the dried RF gels to a large extent, and the value for the Young's moduli may vary compared to reported samples.^26^

CAs containing hollow spheres are obtained with a much larger reversible deformation ability with over 50% compressive strain compared to pristine ones. Fig. 3 shows the collapse of the pristine sample at 30% compressive strain, contrary to the sample containing hollow PS spheres. The destruction of the pristine sample is accompanied by fragmentation and irreversible damage during compression. A picture sequence of the compression test in the initial, maximal compression and the final released stage is shown in ESI, Fig. S8 and Video 1 and 2.† We conclude that the deformation of macropores with a uniform size under compression is responsible for the improvement of the flexibility and mechanical stability.

In agreement with the literature,^7^ we observed a strong increase of the surface area for the carbon aerogel in comparison to the RF gel (Table 1). In the case of PS addition we obtained only a slightly higher surface area with 661 m^2^ g^−1^ compared to the pristine one (577 m^2^ g^−1^), while the pore volume is not affected (0.4 cm^3^ g^−1^ (ESI, Fig. S9†)). The pore size distributions reveal for both cases a hierarchically structured material containing micro-, meso- and macropores.

**Fig. 4:**
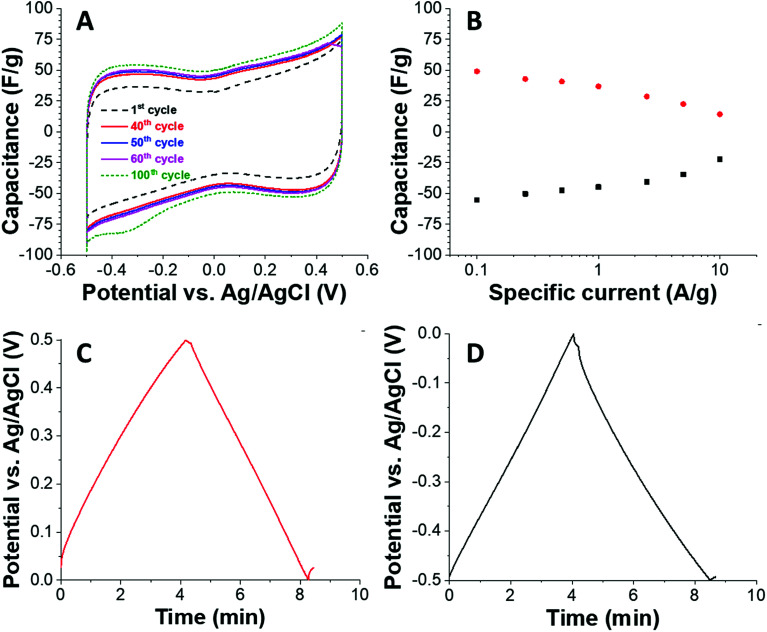
Electrochemical characterization of C + PS in half-cell configuration using aqueous 1 M NaCl. (A) Cyclic voltammetry recorded at 1 mV s^−1^. (B) Galvanostatic charge/discharge at different specific currents (rate handling). (C and D) Galvanostatic charge/discharge profiles recorded at 0.1 A g^−1^.

Based on the high surface area and the intrinsic electrical conductivity of carbon, we also tested the material with the highest porosity (C + PS) as an electrode material for electrical double-layer capacitors. We tested the electrochemical properties in an aqueous 1 M NaCl because of the possible application for water desalination *via* capacitive deionization and because of the environmental friendliness of this kind of electrolyte. Using a half-cell geometry with an oversized activated carbon counter electrode, we see a rather rectangular shaped cyclic voltammogram (Fig. 4A) at a sweep rate of 1 mV s^−1^. Due to progressively improved wetting of the carbon, we see an increase in capacitance when comparing the 1^st^ and the 100^th^ charge/discharge cycle. For a better quantitative analysis of the capacitance, we used galvanostatic charge/discharge profiling (Fig. 4B–D). At a low specific current of 0.1 A g^−1^, we obtained about 50 F g^−1^ for positive polarization (49 F g^−1^) and negative polarization (55 F g^−1^) with a coulombic efficiency nearing 100%. When increasing the specific current, the capacitance decreases and because of the hierarchic porous system we maintain 63% and 46% of the initial energy storage capacity at 5° A g^−1^.

The energy storage capacity depends on the use of electrolyte, and we see often enhanced values in acidic aqueous media when there are redox-active surface groups at the carbon/electrolyte interface.^27^ For nanoporous carbons used as electrodes for electrical double-layer capacitors, there is a strong influence of the pore size on the energy storage capacity and the latter is limited by the total available and ion-accessible surface area. In case of C + PS, the specific surface area is 661 m^2^ g^−1^, which is much lower than many commercially available activated carbons with values of 1500–2500 m^2^ g^−1^. Accordingly, we measure a specific capacitance of about 50 F g^−1^, corresponding with a value of about 0.08 F m^−2^. For example, the often-used activated carbon YP80F of Kuraray has a specific surface area of 1786 m^2^ g^−1^ and accomplishes in the same electrolyte as used in our present work (1 M NaCl) an energy storage capacity of about 130 F g^−1^; this corresponds with an area normalized capacitance of 0.07 F m^−2^.^28^ As another example, Kynol 507-20 activated carbon has a specific surface area of 1940 m^2^ g^−1^ and accomplishes in 1 M NaCl aqueous electrolyte a capacitance of 140 F g^−1^ (= 0.07 F m^−2^).^29^ Therefore, we see a solid electrochemical performance of carbon C + PS which is limited only by the overall moderate surface area.

## Conclusions

We introduced a route with high potential to prepare various kinds of reversibly compressible carbon aerogels with a tailored, hierarchical pore structure, compression modulus and even increased ultimate strength. Furthermore, we expect with our synthetic route a high potential to introduce new functionalities into the carbon network by PS templating. The covalent fixation is indicated by the lack of PS dissolution with acetone prior and during supercritical drying. The incorporation of hollow spheres can serve on the one hand as a model system to understand the origin for its flexibility and on the other properties such as high electrical conductivity, high pore volume, and compressibility, make them interesting candidates also for electrochemical applications.

## Conflicts of interest

The authors declare no conflict of interest.

## Supplementary Material

RA-008-C8RA04848G-s001

RA-008-C8RA04848G-s002

RA-008-C8RA04848G-s003
