# Supplementary material for: Carbon aerogels with improved flexibility by sphere templating
Source: RSC Adv. 2018 Jul 31;8(48):27326–31. doi: 10.1039/c8ra04848g (PMC9083318; doi:10.1039/c8ra04848g)
Supplement: RA-008-C8RA04848G-s003 [file RA-008-C8RA04848G-s003.pdf]

Electronic Supplementary Material (ESI) for RSC Advances.
This journal is © The Royal Society of Chemistry 2018

Supporting Information:

Carbon Aerogels with Improved Flexibility by Sphere Templating

Miralem Salihovic,[a] Nicola Hüsing,[a] Johannes Bernardi,[b] Volker Presser,[c,d] and Michael S. Elsaesser[a*]

[a] *Chemistry and Physics of Materials, University of Salzburg, 5020 Salzburg, Austria.*
[b] *USTEM, Vienna University of Technology, 1040 Vienna, Austria.*
[c] *INM – Leibniz Institute for New Materials, 66123 Saarbrücken, Germany*
[d] *Saarland University, 66123 Saarbrücken, Germany*
[*] *Corresponding author. Tel: +43-662-8044-6262. E-mail: michael.elsaesser@sbg.ac.at (Michael S. Elsaesser)*

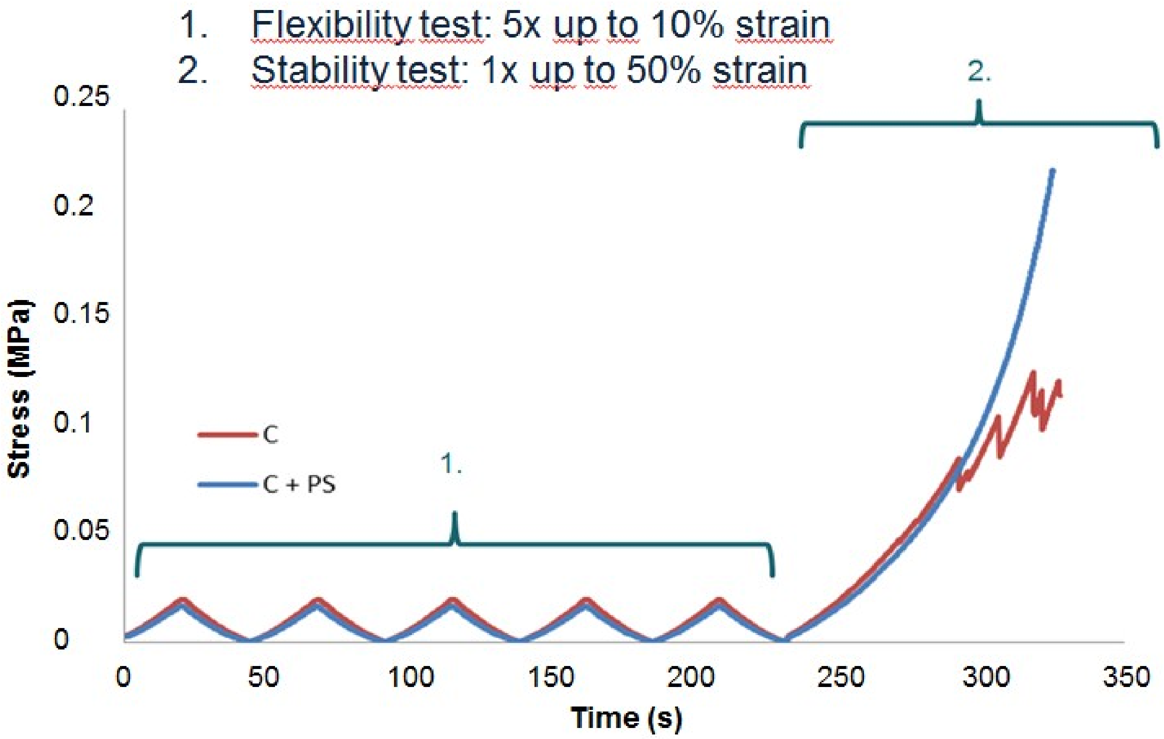

Figure S1. Chronological succession of the flexibility and stability tests during the mechanical testing experiments.

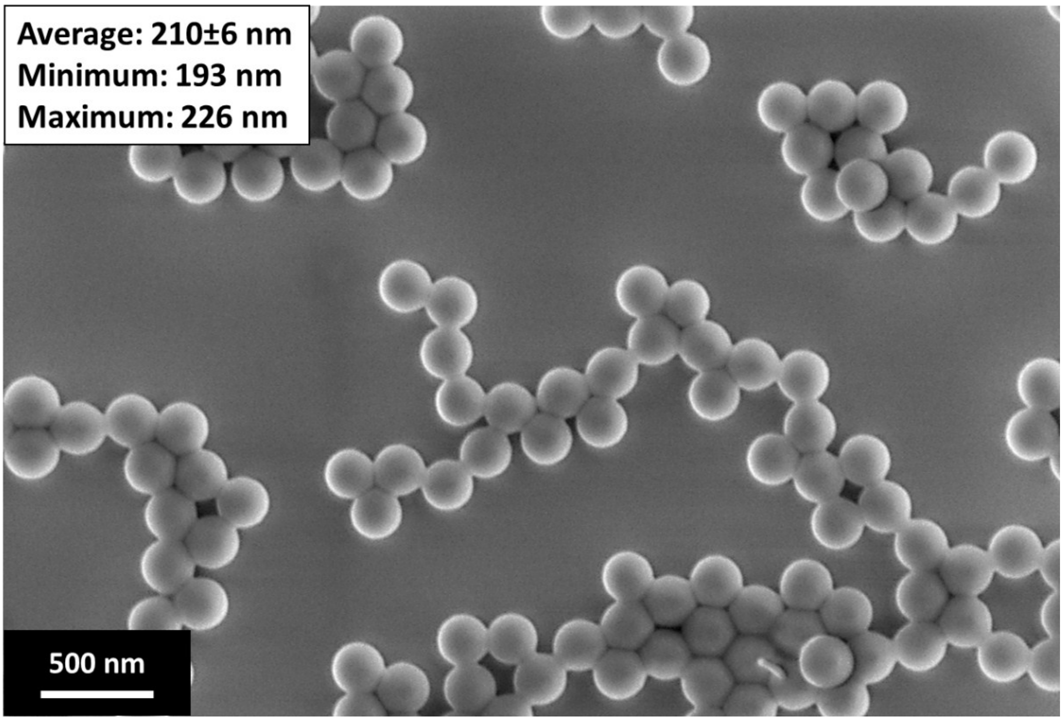

Figure S2. Scanning electron micrograph of the polystyrene nanospheres. The size data relate to measurements of 100 individual nanospheres.

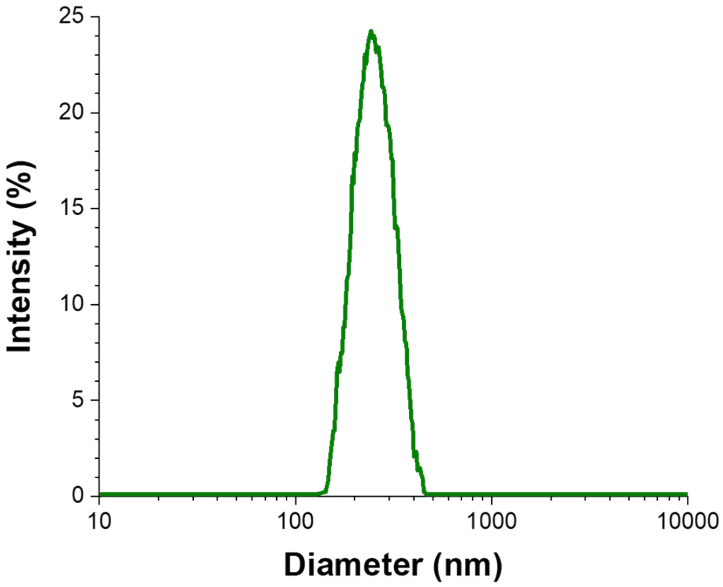

Figure S3. Size distribution of PS nanospheres obtained from dynamic light scattering.
Z-average = 244 nm, PdI = 0.020.

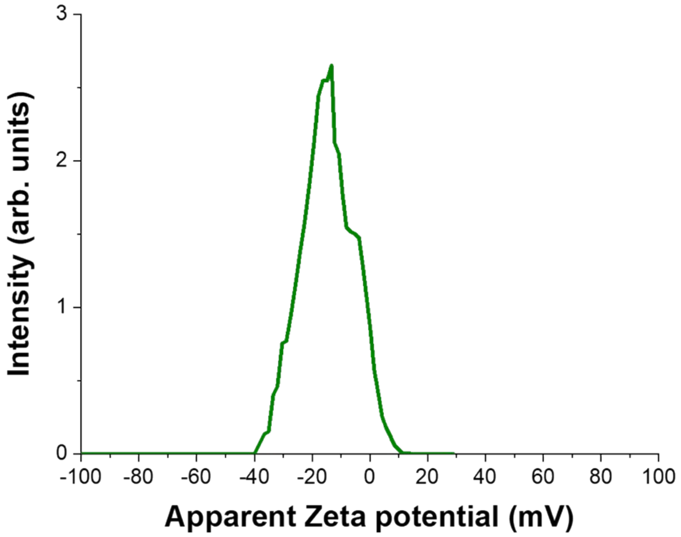

Figure S4. Zeta potential of PS nanosphere solution yielding an average value of -15.7 mV from 12 runs.

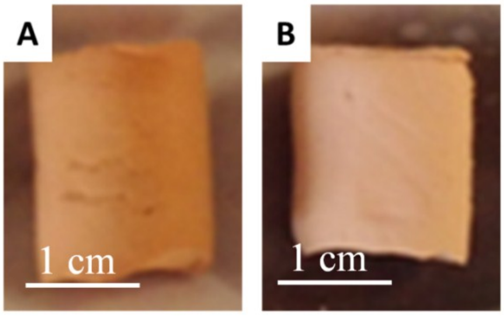

Figure S5. Photographs of (A) pristine RF aerogel, (B) RF + 0.1 g PS.

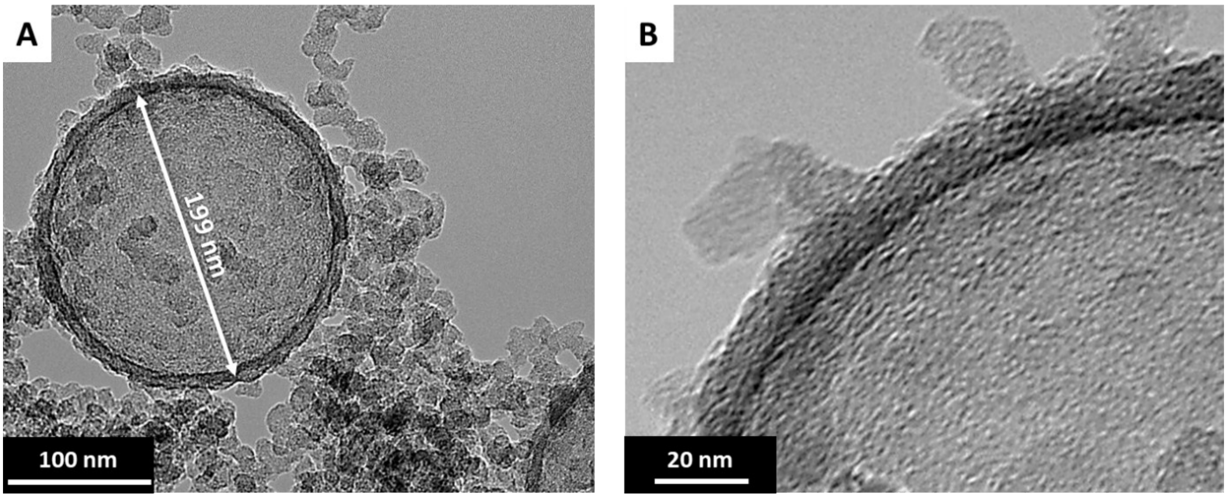

Figure S6. Transmission electron micrographs of a hollow sphere found in C+PS.

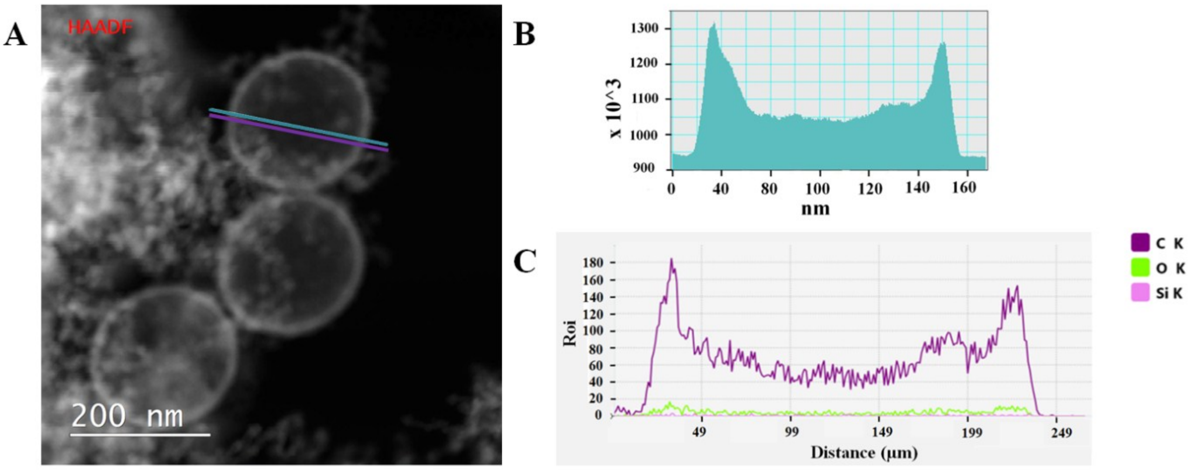

Figure S7. (A) High angle annular dark field (HAADF) scanning transmission electron microscopy image (STEM), (B) intensity scan according to the turquoise line in (A), energy dispersive X-ray spectra (EDX) for C, O and Si according to the violet line in (A).

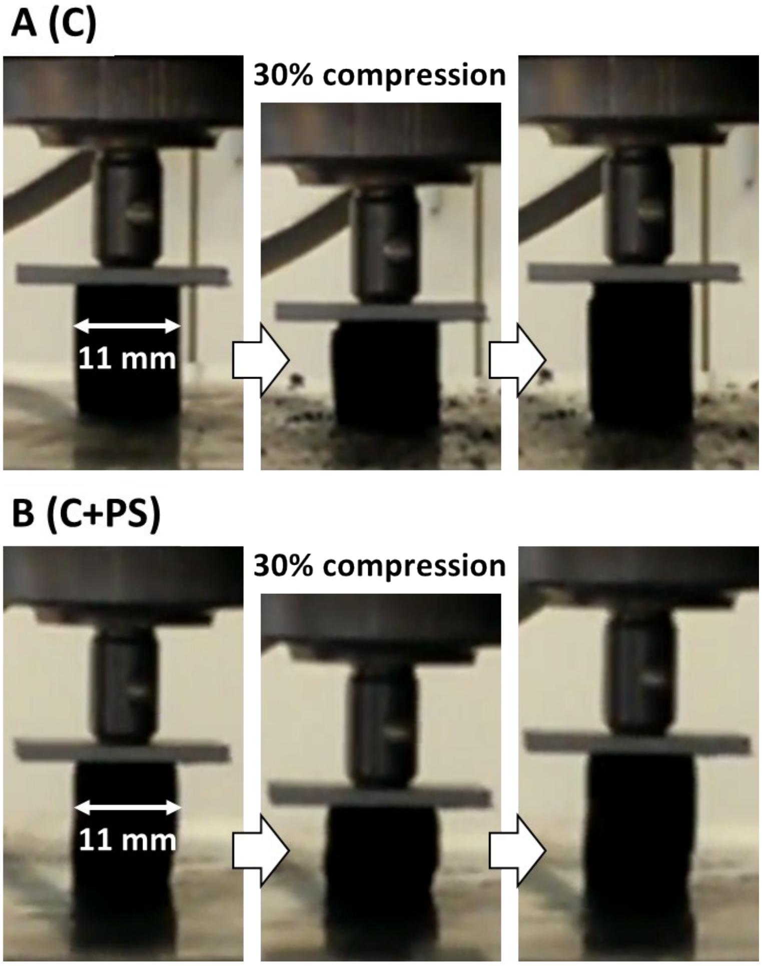

Figure S8. Photographs during mechanical testing. (A) Pristine flexible C aerogel monolith and (B) C+PS aerogel.

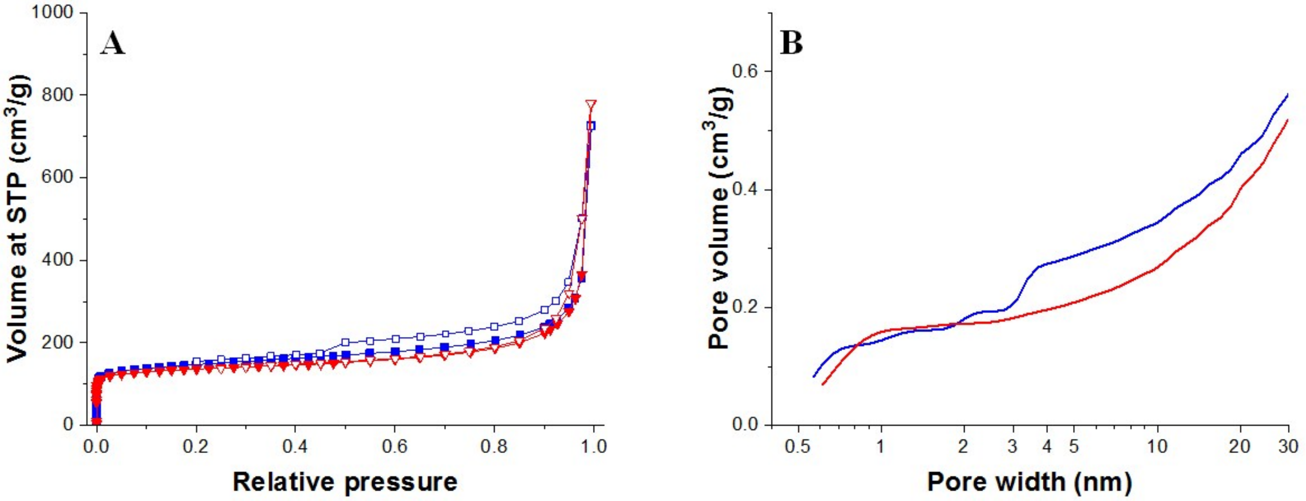

Figure S9. Typical nitrogen adsorption/desorption isotherms (A) and pore size distributions (QSDFT) (B) for a pristine flexible CA (red) and a PS modified CA (blue).
